# Ebola Virus Disease Cases Among Health Care Workers Not Working in Ebola Treatment Units — Liberia, June–August, 2014

**Published:** 2014-11-21

**Authors:** Almea Matanock, M. Allison Arwady, Patrick Ayscue, Joseph D. Forrester, Bethany Gaddis, Jennifer C. Hunter, Benjamin Monroe, Satish K. Pillai, Christie Reed, Ilana J. Schafer, Moses Massaquoi, Bernice Dahn, Kevin M. De Cock

**Affiliations:** 1 Epidemic Intelligence Service, CDC; 2 United States Agency for International Development, Liberia; 3 Division of High-Consequence Pathogens and Pathology; 4 Division of Preparedness and Emerging Infections, National Center for Emerging and Zoonotic Infectious Disease, CDC; 5 President’s Malaria Initiative, Center for Global Health, CDC; 6 Division of Epidemiology, Analysis, and Library Services, Center for Surveillance, Epidemiology, and Laboratory Services, CDC; 7 Clinton Health Access Initiative; 8 Liberian Ministry of Health and Social Welfare; 9 CDC Kenya, Center for Global Health, CDC

West Africa is experiencing the largest Ebola virus disease (Ebola) epidemic in recorded history. Health care workers (HCWs) are at increased risk for Ebola. In Liberia, as of August 14, 2014, a total of 810 cases of Ebola had been reported, including 10 clusters of Ebola cases among HCWs working in facilities that were not Ebola treatment units (non-ETUs). The Liberian Ministry of Health and Social Welfare and CDC investigated these clusters by reviewing surveillance data, interviewing county health officials, HCWs, and contact tracers, and visiting health care facilities. Ninety-seven cases of Ebola (12% of the estimated total) were identified among HCWs; 62 HCW cases (64%) were part of 10 distinct clusters in non-ETU health care facilities, primarily hospitals. Early recognition and diagnosis of Ebola in patients who were the likely source of introduction to the HCWs (i.e., source patients)* was missed in four clusters. Inconsistent recognition and triage of cases of Ebola, overcrowding, limitations in layout of physical spaces, lack of training in the use of and adequate supply of personal protective equipment (PPE), and limited supervision to ensure consistent adherence to infection control practices all were observed. Improving infection control infrastructure in non-ETUs is essential for protecting HCWs. Since August, the Liberian Ministry of Health and Social Welfare with a consortium of partners have undertaken collaborative efforts to strengthen infection control infrastructure in non-ETU health facilities.

Human-to-human transmission of Ebola virus occurs through direct contact with the body fluids of symptomatic or deceased patients. HCWs in Liberia working without adequate infection control equipment and protocols are at high risk for infection given their close physical contact with Ebola patients and potential exposure to body fluids. HCWs have accounted for up to 25% of infected persons during previous outbreaks (1). Isolating infected patients is essential for preventing transmission to others, and historically this has been accomplished by caring for infected persons in specialized ETUs with strict isolation and infection control protocols, including guidelines for patient movement, physical layout, disinfection, and use of PPE designed to protect HCWs and patients (2,3). Ideally, all patients suspected of having Ebola would be triaged and tested at an ETU (1); however, before recognition of Ebola and transfer to an ETU, infected patients often are cared for in non-ETU health care facilities. Treatment of Ebola in non-ETU health care facilities is particularly difficult in Liberia, where the health care system is understaffed and under-resourced (4). Visits to non-ETU health care facilities revealed that basic materials for standard infection control practices such as gloves, soap, and water often were inadequate, and overcrowding in patient care areas plus the lack of physically separated spaces made isolation difficult. Because Ebola is a febrile illness with nonspecific signs and symptoms, differentiating it from many other common febrile illnesses is difficult, potentially delaying isolation.

As of August 14, 2014, a total of 810 confirmed, probable, and suspected cases of Ebola† in six of Liberia’s 15 counties had been reported (5). There were two primary epicenters in Liberia: Lofa County in northwestern Liberia, where the outbreak in Liberia was initially detected following movement of infected persons over the border from Guinea; and Montserrado County, which includes the capital city of Monrovia (Figure). Because of the scale and geographic distribution of the outbreak, the lack of staff, beds, and transportation to ETUs, as well as patient resistance to being treated in ETUs, only an estimated 25% of known Ebola patients had been treated at an ETU as of August 14, 2014 (5). At the request of the Liberian Ministry of Health and Social Welfare, CDC collaborated with the ministry to investigate risks associated with working in health care settings and possible sources of exposure among HCWs.

Reviews were performed of national surveillance data, including case report forms, health care facility line lists, the national surveillance database, and laboratory results. Clusters were defined as two or more confirmed, probable, or suspected cases of Ebola among HCWs who had dates of symptom onset or, when symptom onset was not available, dates of diagnosis within 21 days of each other and any subsequent chains of transmission. Source patients were identified prospectively in some clusters, and retrospectively in others. Evaluations of the recognized clusters of HCWs were performed using unstructured in-person and telephone interviews with county health officials, hospital staff members, and contact tracers, as well as visits to six of the 10 health facilities with identified clusters of Ebola among HCWs. HCW cases of Ebola not identified as part of the clusters and risk factors outside of health care settings were not evaluated. No patient care was directly observed.

Review of national case-based surveillance data and field investigations of clusters of Ebola in HCWs through August 14 identified 97 HCWs with Ebola. Among the 97 HCW cases, the most common occupation was nurse or nurse aide (35%), followed by physician or physician assistant (15%); other occupations included laboratory technicians, cleaners and hygienists, administrators, midwives, dispensers, and security personnel (Table 1). Most of these Ebola cases occurred in HCWs employed at hospitals (60%). However, all types of health care settings (including public and private) experienced cases of Ebola among HCWs, from the smallest clinics, which have catchment areas of <3,500 persons and are open Monday through Friday without inpatient services, to larger regional hospitals, which have catchment areas of three to five counties and are expected to be open 24 hours a day with at least a 100-bed capacity (6).

Among the 97 HCW cases, 11 clusters of Ebola occurred (10 in non-ETU facilities and one in an ETU) during June 9–August 14 in four counties (Bong, Lofa, Margibi, and Montserrado) (Figure). The one cluster involving HCWs who worked primarily in an ETU and triaged patients from an associated hospital has been described previously (7). Among the remaining 10 clusters that occurred in non-ETU health care facilities, the number of cases ranged from two to 22 HCWs per cluster (median = five HCWs). Included in these 10 clusters were 62 (64%) of the 97 HCWs with Ebola identified overall (Table 2). Of the 62, a total of 50 (81%) had confirmed Ebola, and 31 were known to have died. Seven of 10 HCW clusters were primarily associated with hospitals. One cluster included HCWs in two clinics and a hospital; a single source patient visited all three locations while ill. The remaining two clusters occurred among HCWs who worked in two separate clinics.

Of the 62 HCWs involved in the 10 clusters, 33 were identified as having cared for the source patient in the cluster. Examples of reported high-risk exposures among the infected HCWs included a spill of infected patient blood onto the uncovered skin of a phlebotomist and medical care provided by HCWs not using adequate PPE when caring for a fellow HCW who was ill with what was thought to be heart failure, but later was diagnosed as Ebola. Additionally, possible high-risk exposure occurred by direct physical contact of two HCWs with an infected patient whom the HCWs had assisted into the hospital. In two of the clusters, the source patients were HCWs who had reportedly cared for infected patients at home, outside of their regular job duties. Four HCWs among three of the 10 clusters had no known or identified unprotected physical contact with patients with Ebola, but worked in health facilities where patients with Ebola had been treated. For example, an HCW who served as the officer-in-charge of an outpatient department was infected. This HCW had no direct contact with the source patient, but had worked closely with many of the HCWs who developed secondary cases.

In four of the 10 clusters, the source patients were suspected of having Ebola when initially examined, based on history and clinical symptoms. However, in four other clusters, the source patient was initially thought to have another disease (e.g., dysentery, cholera, Lassa fever, or heart disease). In one of these four clusters, the source patient had a known history of heart disease and did not disclose a history of Ebola virus exposure leading to a delay in diagnosis. In another cluster, details of testing are unclear, but the source patient was not confirmed to have Ebola virus until at least 12 days after developing symptoms. Of the remaining two clusters, a source patient could not be identified in one cluster, and investigation of the other was incomplete because five HCWs had died and the health facility director could not be contacted

Visits to six of the 10 non-ETU health care facilities where clusters occurred revealed that materials and setup required for implementing adequate infection control precautions often were not available. These included adequate chlorine, running water, cleaning supplies, hand washing stations, adequate types and supplies of PPE, and isolation areas. In instances where limited PPE was available, equipment was shared or reused. At one hospital visit, it was reported that multiple HCWs consecutively donned and doffed the same pair of single-use gloves to care for a patient with Ebola. Alternatively, some HCWs were noted to be wearing the same PPE throughout their shift while caring for Ebola and non-Ebola patients. Isolation areas existed at five of the six health facilities visited where there were clusters of Ebola among HCWs, but were inadequate. For example, at one hospital, a single occupancy room within the emergency department was used for isolation but was quickly overwhelmed when the facility admitted multiple patients with Ebola in a week. The isolation areas were rudimentary, lacking toilet facilities, running water, and physical separation from other patient treatment areas.

## Discussion

These infections demonstrate the risk associated with caring for Ebola patients without adequate infection control. Individual cases and clusters of Ebola continued to occur among HCWs working in non-ETU health care facilities in Liberia during the period covered by this investigation, reflecting ongoing transmission and the increasing burden of Ebola in the community. Nurses and nurse aides were most commonly infected, although cases of Ebola among HCWs in all occupations, both clinical and nonclinical, were observed. By early August, many of the health care facilities in Liberia were either functionally or officially closed because of inability to maintain staffing as a result of HCW illnesses and departures and patient avoidance of facilities where Ebola patients had been treated.

Inadequate infection control infrastructure, including inadequate protocols, training, materials, and setup contributed to Ebola virus exposure in the non-ETU health care settings described in this report. Supplies of PPE were insufficient across Liberia and, when available, often were not adequate or improperly used. During the course of this investigation, many health care facilities closed; however, preparation for reopening closed health facilities was under way, including training for infection prevention and control. As conditions of reopening, HCWs not only requested training, but also a consistent supply of adequate PPE.

Early recognition, triage, and isolation of all potential Ebola cases are essential so that adequate infection control measures can be applied and transmission of Ebola virus limited. Ebola symptoms are similar to those of many other diseases, and recognition is difficult when not initially suspected. In Liberia, Ebola should be considered in all patients with fever or other symptoms because of 1) the relatively high incidence of the disease; 2) ongoing opportunities for acquisition through direct contact with body fluids of symptomatic or deceased patients during patient care, handling of a dead body, or environmental contact with body fluids; 3) variable reliability of patient reports of their risk factors; and 4) difficulties in contact tracing, including limited availability and timeliness of laboratory testing. After triaging possible cases, patients should be isolated with adequate infection control measures (3). As demonstrated in these clusters, inaccurate illness and exposure histories and difficulties in making a clinical diagnosis can result in additional exposures. These factors make it critical that all HCWs, both clinical and nonclinical, who might encounter infected patients or contaminated environments or materials, have access to and adhere to infection control measures.

What is already known on this topic?

Human-to-human transmission of Ebola virus disease (Ebola) can occur through direct contact with body fluids of symptomatic or deceased patients. Health care workers (HCWs) are at greater risk for Ebola, accounting for up to 25% of cases in previous outbreaks. These risks can be mitigated by triage protocols, adherence to strict infection control guidelines, and adequate provisions and use of personal protective equipment. Strong infection control is essential to breaking the chain of transmission of Ebola virus.

What is added by this report?

During June 9–August 14, 2014, a review of national data and field investigations identified 97 cases of Ebola among HCWs in Liberia, 62 of which occurred in 10 clusters in health care facilities not dedicated to treating Ebola patients, primarily hospitals. Individual cases and clusters of Ebola among HCWs occurred most often among nurses, nurse aides, and physicians. However, there were cases of Ebola among HCWs in all occupations and health care settings. Infrastructure for adequate infection control was lacking.

What are the implications for public health practice?

To avoid the acquisition of Ebola among HCWs, especially in the health care setting, and the subsequent undermining of the epidemic response, a strong infection control infrastructure is needed. Working towards this, the Liberian Ministry of Health and Social Welfare in collaboration with a consortium of partners has initiated a major program to improve infection prevention and control at health care facilities. This program emphasizes rapid recognition and triage, appropriate training in the use of and adequate supply of personal protective equipment, and identification of a structure for the supervision of consistent and appropriate infection control adherence.

Direct physical contact with the body fluids of infected patients while at work continues to be a clear risk factor, but exposures outside the health care setting also were noted (i.e., the two HCW source patients who had cared for infected patients at home). With many facilities closed and ongoing community transmission, HCW risks for acquiring Ebola in the community exist. Additionally, although no HCW-to-patient or patient-to-patient transmissions were identified because this investigation was limited to infected HCWs, patients likely also had direct physical contact with other patients and environmental exposures to Ebola virus in these health care settings.

The findings in this report are subject to at least four limitations. First, collection of data on exposure history and infection control practices was limited by deaths and illness among HCWs from Ebola (31 deaths at the time of the investigation, with other HCWs critically ill), a lack of coworker proxies to provide history for many of the cases, and the closure of health facilities, which made it difficult to locate HCWs. Second, infection control practices were not systematically observed, and reports might have been affected by recall bias. Third, exposure histories were difficult to evaluate because multiple cases of Ebola were treated simultaneously by individual HCWs and there also was the potential for environmental exposure in the work place and community exposures. Finally, evaluation of exposure and disease transmission contacts was limited by the lack of contact lists in eight clusters and incomplete contact lists in the other two.

The immediate consequences of Ebola among HCWs, especially when occurring in clusters at individual facilities, are the closure of health facilities, loss of routine services, grief and fear among HCWs, and public mistrust of HCWs and health facilities, all of which might undermine the epidemic response. The long-term consequences include the loss of a sufficient and experienced HCW work force to provide health services and educate future HCWs. Both the immediate and long-term consequences are likely to result in increased non-Ebola morbidity and mortality.

Effective isolation is at the core of a robust Ebola response and cannot be performed without strong infection control in a functioning health care system. Strong infection control is essential to breaking the chain of transmission of the Ebola virus, which is necessary in reestablishing routine health care in Liberia. To begin to accomplish this, there needs to be recognition and triage of potential cases of Ebola, appropriate training in the use of and adequate supply of personal protective equipment, and identification of a structure for the supervision of consistent infection control adherence.

Since August, collaborative efforts to strengthen infection control infrastructure in non-ETU health facilities have been undertaken by a consortium of partners working with the Liberian Ministry of Health and Social Welfare. These efforts included developing national guidance for infection control standards necessary to deliver health services. A training program on infection control, including triage and isolation of suspected Ebola cases, appropriate use of PPE, and environmental hygiene, has been initiated for HCWs of all occupational types working in all levels of the health care system throughout Liberia. Importantly, a culture of infection prevention will be emphasized by identifying infection control specialists who will be embedded in non-ETU health facilities to supervise adherence to infection control practices. These efforts to implement, assess, and improve infection control in non-ETU health care settings are an ongoing and essential component of the response.

## Figures and Tables

**FIGURE:**
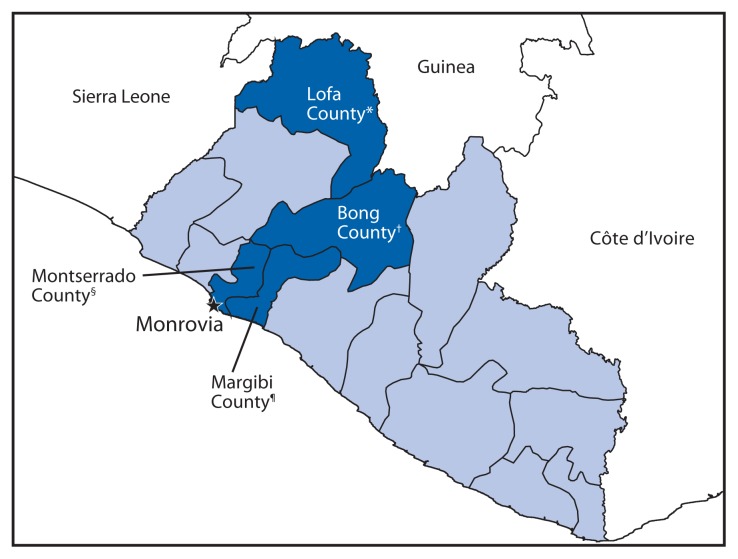
Counties of Liberia where clusters of Ebola virus disease were reported among health care workers in health care facilities that were not Ebola treatment units — June 9–August 14, 2014 * Three clusters with 12 total cases (nine confirmed). ^†^ One cluster with five total cases (five confirmed). ^§^ Five clusters with 23 total cases (19 confirmed). ^¶^ One cluster with 22 total cases (17 confirmed).

**TABLE 1:** Number of cases (suspected, probable, and confirmed) of Ebola virus disease among persons identified as health care workers, by occupation and type of facility where workers were employed — Liberia, June 9–August 14, 2014*

Occupation/Facility	No.	(%)
**Occupation**		
Nurse	23	(24)
Nurse aide	11	(11)
Physician	10	(10)
Laboratory technician	8	(8)
Physician assistant	7	(7)
Cleaner/Hygienist	5	(5)
Dispenser	3	(3)
Health or surveillance officer	3	(3)
Midwife	3	(3)
Clergy	2	(2)
Vaccinator	2	(2)
Administrator	1	(1)
Security	1	(1)
Unknown	18	(19)
**Total**	**97**	**(100)**
**Facility**		
Hospital	58	(60)
Clinic	19	(20)
Ebola treatment unit	3	(3)
Health center	1	(1)
Mobile clinic	1	(1)
Public health office	1	(1)
Unknown	14	(14)
**Total**	**97**	**(100)**

* Information on health care worker occupations and facilities was compiled from health care cluster investigations and the Liberian Ministry of Health national Ebola surveillance system.

**TABLE 2:** Characteristics of identified clusters of Ebola virus disease among health care workers in health care facilities that were not Ebola treatment units — Liberia, June 9–August 14, 2014

Characteristic	No.
**Total number of cases**	**62**
Confirmed cases (Deaths)	50 (31)
Health care workers per cluster	2–22 (median = 5)
Clusters in health care facilities that were not Ebola treatment units	10
Hospitals with a cluster of Ebola among health care workers	8
Clinics with a cluster of Ebola among health care workers	4

## Footnotes

* Source patient is defined as the initial patient in a chain of transmission within the health facility. These include a patient whose Ebola infection was unsuspected (patient zero) and a patient whose Ebola infection was suspected (index case) on admission.

† Case definitions available at http://www.who.int/csr/resources/publications/ebola/ebola-case-definition-contact-en.pdf?ua=1.
